# The Implementation of Diagnostic Assessment in Breast Lump Cases: A Cross-Sectional Study in Sragen, Indonesia

**DOI:** 10.7759/cureus.45841

**Published:** 2023-09-24

**Authors:** Toddy Guntersah, Yufi K Astari, Hanggoro T Rinonce, Susanna H Hutajulu, Diah A Puspandari

**Affiliations:** 1 Department of Pathological Anatomy, Soehadi Prijonegoro Public Hospital, Sragen, IDN; 2 Department of Internal Medicine, Division of Hematology and Medical Oncology, Dr. Sardjito General Hospital, Yogyakarta, IDN; 3 Department of Anatomical Pathology, Faculty of Medicine, Public Health and Nursing, Universitas Gadjah Mada/Dr. Sardjito General Hospital, Yogyakarta, IDN; 4 Department of Internal Medicine, Division of Hematology and Medical Oncology, Faculty of Medicine, Public Health and Nursing, Universitas Gadjah Mada/Dr. Sardjito General Hospital, Yogyakarta, IDN; 5 Department of Health Policy and Management, Faculty of Medicine, Public Health and Nursing, Universitas Gadjah Mada, Yogyakarta, IDN

**Keywords:** indonesia, diagnostic errors, breast neoplasms, breast lump, triple assessment

## Abstract

Introduction

Triple assessment, consisting of clinical breast examination, breast imaging, and fine-needle aspiration biopsy (FNAB), is the gold standard for breast lump diagnosis to avoid diagnostic errors. However, current diagnostic practices for breast lump cases in Indonesia are widely variable and evidence for triple assessment implementation is lacking. We aimed to explore the implementation of diagnostic assessments in breast lump cases, its influencing factors, and its association with diagnostic error.

Methods

This cross-sectional study consecutively recruited 364 females with breast lumps who underwent surgery in Soehadi Prijonegoro Public Hospital, Sragen, Indonesia. Data were retrospectively collected from patient’s medical records. Diagnostic assessments were classified as single assessment with clinical breast examination, double assessment with clinical breast examination and breast ultrasonography (USG) or fine-needle aspiration biopsy (FNAB), and triple assessment. Diagnostic error was defined as a discrepancy between pre- and post-surgery diagnosis or repeated surgery without neoadjuvant chemotherapy. Factors associated with diagnostic assessment implementation, diagnostic error, and repeated surgery were analyzed using the chi-square test.

Results

The choice of diagnostic assessment was influenced by patients’ age and health insurance (p<0.001). Triple assessment was only implemented in 21 (5.8%) breast lump cases. It was more frequently applied in patients ≥40 years (57.1%) and patients with contributory health insurance (76.2%). Diagnostic errors were observed in 84 cases (23.1%) and 47 patients out of them (47%) experienced repeated surgery. The implementation of diagnostic assessments was not associated with diagnostic error (p=0.257) but was significantly associated with repeated surgery in breast cancer (p<0.001). Repeated surgery rates were significantly lowered in cases receiving double assessment with FNAB (p<0.001).

Conclusions

The implementation of triple assessment in the local setting was very low. The choice of diagnostic assessment was influenced by patients’ age and health insurance. Further, double assessment applying clinical breast examination and FNAB significantly decreased repeated surgery rates and thus may serve as an alternative to triple assessment in the limited resource setting.

## Introduction

Breast cancer is the most common type of cancer and the leading cause of cancer deaths among women, worldwide [1]. In Indonesia, data from GLOBOCAN 2020 showed that breast cancer incidence is 65,858 cases with a mortality rate of 9.6%. Breast cancer accounts for 16.6% of all cancers in women and men [2]. The most common symptom of breast cancer is a breast lump. Meanwhile, a breast lump has many differential diagnoses, both malignant and benign lesions including abscess, lipoma, fibrocystic lesion, and fibroadenoma which requires an adequate diagnostic assessment [3].

Triple assessment, consisting of clinical breast examination, breast imaging (mammography or ultrasonography), and fine-needle aspiration biopsy (FNAB), is the gold standard for breast lump diagnosis with high sensitivity and specificity (100% and 99.3%) [4]. The implementation of triple assessment is very essential since deficiency in the diagnostic process can lead to diagnostic errors, which significantly increase morbidity, suboptimal treatment, repeated surgery, and medical costs [5]. Diagnostic error is one of the patient safety parameters along with medication or surgery site error and nosocomial infection. However, diagnostic error is still rarely studied, including in the Indonesian population [6].

Sragen is a district in Central Java province. Central Java province is a province with the third largest population (12.9%) in Indonesia [7]. This province is among the 10 provinces with the highest cancer prevalence in the country having a higher cancer rate than the national rate (2.1 vs. 1.8 per million) [8]. Located in the central part, Sragen is close to many areas both in Central Java province and the neighboring provinces. The Indonesian health insurance administration body has a tiered referral system that facilitates cases with breast lump to be firstly referred to type B or C hospitals for diagnostic and treatment workup. Later, they also can be referred to type A or top referral hospitals for more complex management [9]. Soehadi Prijonegoro Public Hospital is a type B hospital in Sragen functioning as a referral hospital for malignancy cases, particularly for those living in Sragen and the surrounding districts. In 2019, there were 283 breast lump cases in Soehadi Prijonegoro Public Hospital, that underwent different types of surgery including incisional biopsy, excisional biopsy, lumpectomy, mastectomy, and lumpectomy followed by mastectomy. During the study period, national health insurance did not accommodate triple assessment in the outpatient setting. Therefore, not all patients with breast lumps could get a triple assessment as their pre-surgery diagnostic workup.

Triple assessment is the ideal diagnostic workup for breast lump cases [10]. However, current diagnostic workup practices for breast lump cases in Indonesia are widely variable despite the existing guidelines both from the international and national levels [10]. One identified factor that influences the implementation of triple assessment for breast lump cases is the patient’s age. A study by Yue et al. reported that only 35% of patients under 25 years and no breast cancer were diagnosed. This study recommended that triple assessment is rarely needed in young patients and is replaced by double assessment consisting of clinical breast examination and breast ultrasonography [11].

Evidence for the implementation of triple assessment in breast lump cases is lacking. This study aimed to explore the implementation of diagnostic assessment in breast lump cases, its influencing factors, and its association with diagnostic error and repeated surgery.

## Materials and methods

Study participants and design

This is a cross-sectional study of patients with breast lumps who underwent definitive surgery in Soehadi Prijonegoro Public Hospital, Sragen, Central Java, Indonesia. Subjects were recruited using a consecutive sampling method. Patients with operable breast lumps who underwent surgery at Soehadi Prijonegoro Public Hospital in the period from January 1, 2018, to December 31, 2019, were enrolled. Patients with bilateral or inoperable breast lump cases, recurrent breast nodules, history of breast incision or core biopsy, or history of neoadjuvant chemotherapy were excluded. From 376 breast lump cases, 364 were included in the present study. This study has been approved by the Medical and Health Research Ethics Committee, Faculty of Medicine, Public Health and Nursing, Universitas Gadjah Mada, Yogyakarta, Indonesia (#KE/FK/0542/EC/2020). All subjects have provided written informed consent upon hospital admission for the use of their data for research purposes.

Data collection

Data were retrospectively collected from patients’ medical records including demographic data (age and health insurance status), specialty of clinician who performed surgery for the breast lump cases, diagnostic assessment, date of surgery, patients’ diagnosis before and after definitive surgery, and medical cost (diagnostic and surgery costs). Age was classified as <40 and ≥40 years based on median value, health insurance status consisted of non-contributory, contributory health insurance (health insurance for poor and near-poor population), and out-of-pocket. The specialty of the clinician who performed the surgery included oncology surgeon, general surgeon, oncology surgery resident, and general surgery resident, which was grouped as (1) oncology and general surgeon and (2) oncology and general surgery resident.

Study outcomes

The primary outcome of this study was the implementation of diagnostic assessment in the breast lump cases. The secondary outcomes included the influencing factors of diagnostic assessment and its association with diagnostic error and repeated surgery. Diagnostic assessments were classified as follows: (a) single assessment with clinical breast examination; (b) double assessment with clinical breast examination and breast ultrasonography (USG); (c) double assessment with clinical breast examination and fine-needle aspiration biopsy (FNAB); and (d) triple assessment with clinical breast examination, USG, and FNAB. Diagnostic error was defined if there is either a discrepancy between pre- and post-surgery diagnosis or repeated surgery indicating a deficiency of guideline-based treatment. Repeated surgery was defined as patients undergoing two consecutive surgeries in less than two months without neoadjuvant chemotherapy.

Statistical analysis

Patients' baseline characteristics and diagnostic assessment implementation were presented as mean and standard deviation (SD) for continuous data and frequency for categorical data. The sensitivity, specificity, positive predictive value (PPV), negative predictive value (NPV), and diagnostic accuracy were calculated individually for each diagnostic assessment compared to the histopathology reports. Factors associated with diagnostic assessment implementation, diagnostic error, and repeated surgery were analyzed using the chi-square test. The results were considered significant if p<0.05. Statistical data analyses were conducted using SPSS software version 25 (Armonk, NY: IBM Corp.).

## Results

Baseline characteristics

**Figure 1:**
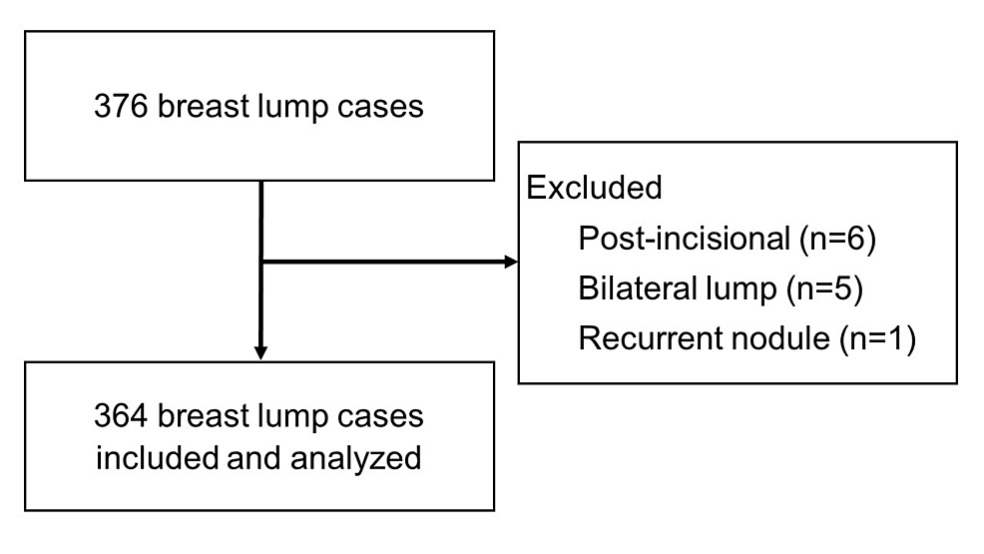
Flow diagram for the included studies.

From 376 breast lump cases, five bilateral cases, one recurrent nodule case, and six post-incisional cases were excluded resulting in 364 cases included in this study (Figure 1). The mean age of patients was 39 years. Most patients had contributory health insurance (52.2%) and were treated by an oncology surgeon (54.7%) (Table 1).

**Table 1:** Characteristics of subjects with breast lump (n=364). Single assessment: clinical breast examination; double assessment: clinical breast examination and breast ultrasonography (USG) or fine-needle aspiration biopsy (FNAB); and triple assessment: clinical breast examination, USG, and FNAB

Characteristics		Frequency (%)
Patients’ age (years) mean±SD		38.90±15.86
≤40 years		190 (52.2)
>40 years		174 (47.8)
Health insurance	Non-contributory health insurance	124 (34.1)
	Contributory health insurance	190 (52.2)
	No health insurance	50 (13.7)
Specialty of clinician	Oncology surgeon	199 (54.7)
	General surgeon	12 (3.3)
	Oncology surgery resident	108 (29.7)
	General surgery resident	45 (12.4)
Diagnostic assessment	Single assessment	163 (44.8)
	Double assessment with USG	75 (20.6)
	Double assessment with FNAB	105 (28.8)
	Triple assessment	21 (5.8)

Diagnostic assessment in breast lump cases

The implementation of triple assessment in included patients was very low (5.8%). In this study, a single assessment with clinical breast examination was the most common diagnostic assessment implemented (44.8%), followed by a double assessment with FNAB (28.2%) and double assessment with breast USG (20.6%).

After assessing each diagnostic assessment performance compared to the histopathology results, triple assessment yielded the highest sensitivity (91.7%), specificity (100%), PPV (100%), and accuracy (95.2%). In the second place, a single assessment with FNAB also demonstrated a high result in sensitivity (83.6%), specificity (90.9%), PPV (92.7%), and accuracy (86.7%). Despite having a high specificity (97%) and accuracy (90.7%), double assessment with breast USG demonstrated a low sensitivity (37.5%) and PPV (60%). Single assessment also showed a low sensitivity (53.1) (Table 2).

**Table 2:** Sensitivity, specificity, positive predictive value, negative predictive value, and accuracy by assessment methods. PPV: positive predictive value; NPV: negative predictive value; FNAB: fine-needle aspiration biopsy

Indicators	Single assessment	Double assessment with USG	Double assessment with FNAB	Triple assessment
Sensitivity (%)	53.1	37.5	83.6	91.7
Specificity (%)	96.9	97.0	90.9	100.0
PPV (%)	80.9	60.0	92.7	100.0
NPV (%)	89.4	92.8	80.0	90.0
Accuracy (%)	88.3	90.7	86.7	95.2

Factors associated with diagnostic assessment implementation in breast lump cases

Patients’ age and health insurance were associated with diagnostic assessment implementation for breast lump cases (p<0.001) (Table 3). The implementation of triple assessment and double assessment with FNAB was higher in patients of age ≥40 years (57.1% and 75.2%), whereas single assessment and double assessment with breast USG were higher in patients under 40 years of age (66.3% and 62.7%).

**Table 3:** Factors associated with diagnostic assessment implementation (n=364). FNAB: fine-needle aspiration biopsy *Expected frequency <5. **Statistically significant p-value.

Variables		Single assessment, n (%)	Double assessment with USG, n (%)	Double assessment with FNAB, n (%)	Triple assessment, n (%)	p-Value
Patients’ age (years)	<40	108 (66.3)	47 (62.7)	26 (24.8)	9 (42.9)	<0.001^**^
	≥40	55 (33.7)	28 (37.3)	79 (75. 2)	12 (57.1)	
Health insurance	Non-contributory	57 (35.0)	23 (30.7)	42 (40.0)	2 (9.5)	<0.001^**^
	Contributory	68 (41.7)	49 (65.3)	57 (54.3)	16 (76.2)	
	Out-of-pocket	38 (23.3)	3 (4.0)	6 (5.7)	3 (14.3)^*^	
Specialty of clinician	Oncology and general surgeon	100 (61.4)	33 (44.0)	65 (61.9)	13 (61.9)	0.056
	Oncology and general surgery resident	63 (38.6)	42 (56.0)	40 (38.1)	8 (38.1)	

The implementation of triple assessment was significantly lowest in patients with non-contributory health insurance (9.5%) and highest in patients with contributory health insurance (76.2%). The implementation of double assessments, both with FNAB and breast USG, was lowest in the out-of-pocket group (5.7% and 4.0%, respectively). Meanwhile, the implementation of a single assessment was quite frequent in the out-of-pocket group (23.3%) and highest in the contributory health insurance group (41.7%). The specialty of the clinician was not significantly associated with diagnostic assessment implementation (p=0.056). However, double assessment with breast USG was more frequent in the resident group (56%).

Association of diagnostic assessment with diagnostic errors and repeated surgery

Diagnostic errors were found in 84 cases (23.1%) and described in Table 4. Among patients with breast cancer diagnosis, 47 patients (47%) experienced repeated surgery. The implementation of diagnostic assessment was not associated with diagnostic error in breast lump cases (p=0.257) but was significantly associated with repeated surgery in breast cancer cases (p<0.001) (Table 5).

**Table 4:** Diagnostic error in breast lump cases (n=84)

Pre-surgery diagnosis	Post-surgery diagnosis	Frequency (%)
Abscess	Ductal carcinoma	1 (1.2)
Fibroadenoma	Abscess	1 (1.2)
Galactocele	Abscess	2 (2.4)
Suspect malignant lesion	Abscess	5 (5.9)
	Carcinoma ductal	30 (35.7)
	Fibroadenoma	2 (2.4)
	Fibrocystic lesion	3 (3.6)
Breast tumor	Abscess	10 (11.9)
	Ductal carcinoma	28 (33.3)
	Malignant phyllodes	2 (2.4)

**Table 5:** Factors associated with diagnostic error in breast lump cases and repeated surgery in breast cancer cases. FNAB: fine-needle aspiration biopsy *Expected frequency <5. **Statistically significant p-value.

Variables		Diagnostic errors (n=364)			Repeated surgery (n=100)		
		Yes	No	p-Value	Yes	No	p-Value
		n (%)	n (%)		n (%)	n (%)	
Patients’ age (years)	<40	25 (29.8)	165 (58.9)	<0.001^**^	9 (19.2)	1 (1.9)	0.004^**^
	≥40	59 (70.2)	115 (41.1)		38 (80.8)	52 (98.1)	
Diagnostic assessment	Single assessment	42 (50.0)	121 (43.1)	0.257	23 (48.9)	0 (0)	<0.001^**^
	Double assessment with USG	11 (13.1)	64 (22.9)		6 (12.8)^*^	0 (0)	
	Double assessment with FNAB	25 (29.8)	80 (28.6)		14 (29.8)	45 (84.9)	
	Triple assessment	6 (7.4)^*^	15 (5.4)		4 (8.5)	8 (15.1)	
Specialty of clinician	Oncology and general surgeon	44 (52.4)	167 (59.6)	0.237	33 (70.2)	42 (79.3)	0.298
	Oncology and general surgery resident	40 (47.6)	113 (40.4)		14 (29.8)	11 (20.7)	

Repeated surgery occurred most frequently in the single assessment group and least frequently in the triple assessment group (48.9% vs. 8.5%). All patients in groups of single assessment and double assessment with breast USG experienced repeated surgery. Breast cancer cases without repeated surgery were mostly diagnosed with a double assessment with FNAB (84.9%).

Meanwhile, after analyzing the other factors, diagnostic errors were found significantly higher in patients of age ≥40 years (p<0.001). In contrast, repeated surgery was significantly lower in patients of age ≥40 years (p=0.004). The specialty of the clinician was not significantly associated either with diagnostic error or repeated surgery (p=0.237 and p=0.298).

## Discussion

This study found that the most common diagnostic assessment in Soehadi Prijonegoro Public Hospital was a single assessment with clinical breast examination (44.8%), followed by a double assessment with clinical examination and FNAB (28.8%). Meanwhile, the triple assessment was only conducted in 5.8% of cases. Evidence for the implementation of diagnostic assessment in breast lump cases is scarce. In developing countries, including Indonesia, there are limited healthcare resources and a lack of financial support that often leads to the implementation of different strategies to diagnose breast lesions [12]. A previous study from Nigeria reported that triple assessment is frequently impractical since imaging is unavailable and core needle biopsy is challenging. As a result, the use of clinical examination and fine-needle aspiration cytology (FNAC) was emerging with imaging components playing a secondary role [13].

National guidelines for breast cancer management state that triple assessment must be conducted for all breast lump cases in patients over 35 years [10]. The implementation of triple assessment in the present study was very low, indicating a non-adherence practice to the existing guideline. This non-adherence phenomenon can be affected by internal (patient’s characteristics, clinician) and external factors (e.g., refusal from patients, lack of facility, lack of supportive environment, and cost-related problems) [14].

The median age of the included patients was 39 years. The implementation of double assessment with FNAB and triple assessment was higher in patients ≥40 years, while double assessment with USG and single assessment were more frequent in younger patients. Previous studies reported that triple assessment might not be urgently needed for all breast lump cases because it is expensive and invasive [11]. Yue et al. in 2015 recommended that biopsy examination in patients younger than 25 years was optional and conducted only if the clinical examination or breast USG showed abnormal results [11].

Patient’s health insurance plays a role in influencing the use of clinical practice guidelines [15]. National health insurance in Indonesia covered 78.5% of participants, consisting of contributory (46.0%) and non-contributory (32.4%) schemes [16]. The implementation of triple assessment is significantly higher in contributory health insurance compared to non-contributory health insurance. In addition, clinical breast examination alone is the most common diagnostic assessment applied for patients with out-of-pocket payment. The national health insurance used in Soehadi Prijonegoro Public Hospital did not cover triple assessment as a pre-operative diagnostic workup in the outpatient setting, which might have caused the low implementation of triple assessment [17]. A recommendation to include triple assessment costs in the outpatient setting in the national health insurance system is needed to increase triple assessment implementation in the type B hospital.

This study found no significant differences in diagnostic assessment implementation among clinicians. However, double assessment with breast USG is more frequently ordered by oncology or general surgery residents. The choice of diagnostic tool modality could reflect the clinician’s adherence to the clinical practice guideline. Adherence to clinical practice guidelines is influenced by clinician’s attitudes (concern about guideline content), usual practices, access to resources, and organizational support. In general, younger clinician is more inclined to use clinical practice guideline than older or more experienced clinician [14,15]. Further investigation of clinician’s characteristics impact on diagnostic assessment implementation is warranted.

This study found no association between diagnostic assessment implementation and diagnostic error. However, there is a significant association between diagnostic assessment implementation and repeated surgery occurrence. Repeated surgery in breast cancer indicates that the patients receive suboptimal treatment. In this study, repeated surgery was found in 47% of breast cancer patients, in line with prior studies that reported repeated surgery rates of 10-60% following initial lumpectomy. Repeated surgery represented an added burden to the patient and healthcare system, and increased medical costs [18]. Among 100 breast cancer patients, all patients in single assessment and double assessment with breast USG groups experienced repeated surgery. In this circumstance, although clinician could differentiate benign and malignant lesions based on their clinical examination and judgment, there was always a chance of having an error [19].

Aside from the diagnostic assessment modality, clinician was found not related to diagnostic error or repeated surgery occurrence. Meanwhile, patients’ age was significantly associated with both diagnostic error and repeated surgery. Diagnostic error was significantly higher in patients of age ≥40 years but repeated surgery was significantly lower. Repeated surgery rates were significantly lower in double assessment with FNAB group. FNAB is a quick, affordable, and highly accurate procedure for diagnosing breast lesions [20]. Our study also demonstrated that double assessment with FNAB had a high result in sensitivity (83.6%), specificity (90.9%), PPV (92.7%), and accuracy (86.7%). Previous studies reported that FNAB was significantly more accurate for diagnosing breast cancer than clinical examination and imaging, and can reduce the need for a second surgery [13,20].

We found that double assessment with breast USG and single assessment groups had the highest medical costs consisting of diagnostic and surgery costs (median of Indonesian Rupiah {IDR} 5,022,072 and IDR 4,398,000, respectively). In addition, triple assessment group has a lower cost (median IDR 4,031,275) and double assessment with FNAB group has the lowest cost (median IDR 3,929,499) (table in Appendix). Due to high accuracy and lower medical costs, double assessment with clinical breast examination and FNAB can be introduced as a suitable alternative to triple assessment.

Prior to our study, a direct comparison of the diagnostic procedures, including single, double, and triple assessment, had not been performed. Data obtained from this study suggested that clinical breast examination and FNAB may be clinically equivalent and less costly than triple assessment. Double assessment with FNAB can be a more suitable workup choice for developing or limited-resources countries.

Limitations

This study has some limitations. Firstly, the implementation of a diagnostic tool might be influenced by the clinician’s characteristics (age, attitudes, usual practices) and organizational support [14,15]. However, these factors have not been covered in this study. Secondly, we have not included other informal healthcare and non-healthcare costs in addition to diagnostic tool and surgery costs (medical cost) to provide an adequate cost-effective analysis.

## Conclusions

The implementation of triple assessment in the local cases with breast lumps was very low. The application of diagnostic assessment was influenced by patients’ age and health insurance. Double assessment with clinical breast examination and FNAB significantly decreased repeated surgery rates and had the lowest medical costs, making this practice suitable as an alternative to triple assessment in a setting with limited resources. The low implementation of triple assessment and the high practice of single assessment need to be further studied. Future exploration should better detail various factors including clinician characteristics and cultural and healthcare system aspects. Research on diagnostic errors and repeated surgery rates in higher referral facilities (type A hospitals) is warranted on the premises where more advanced equipment and wider insurance coverage are available. A multicenter study may provide data on whether a triple assessment is indeed possible to implement. If so, as a consequence, national health insurance should cover this in type B and C hospitals. Otherwise, a more feasible local guideline is needed to set the implementation of double assessment over single assessment for breast lump diagnostic workup.

## Appendices

**Table 6:** Medical cost comparison across diagnostic assessments (n=100). FNAB: fine-needle aspiration biopsy

Diagnostic assessment	Diagnostic cost (median)	Surgery cost (median)	Total (median)
Single assessment	IDR 30,000	IDR 4,368,000	IDR 4,398,000
Double assessment with USG	IDR 180,000	IDR 4,831,769	IDR 5,022,072
Double assessment with FNAB	IDR 380,000	IDR 3,609,166	IDR 3,929,499
Triple assessment	IDR 530,000	IDR 3,723,418	IDR 4,031,275
